# Case Report: Effect of testicular adenomatoid tumors on testicular endocrine function in children

**DOI:** 10.3389/fendo.2025.1545449

**Published:** 2025-05-27

**Authors:** Yan Li, Junfeng Zhao, Yi Chen, Jing Zhang, Hehe Chen

**Affiliations:** ^1^ Department of Pediatrics Surgery, Women and Children’s Hospital of Ningbo University, Ningbo, Zhejiang, China; ^2^ Department of Surgical Nursing, Women and Children’s Hospital of Ningbo University, Ningbo, Zhejiang, China; ^3^ Department of Intensive Care Medicine, Women and Children’s Hospital of Ningbo University, Ningbo, Zhejiang, China

**Keywords:** adenomatoid tumor, testis function, adolescents, child, serum inhibin B, serum AMH

## Abstract

Adenomatoid tumors, rare benign mesothelial neoplasms primarily involving the reproductive tract, account for 30% of paratesticular masses and predominantly localize to the epididymal head. Although typically diagnosed in adults (mean age: 33 years), pediatric cases are exceptionally rare. Conservative excision with testicular preservation is optimal, yet diagnostic ambiguity often leads to unnecessary orchidectomy. A 5.2-year-old boy presented with asymptomatic left testicular enlargement. MRI identified a 13 × 14 mm heterogeneous mass on the lateral testis. Intraoperative frozen section analysis suggested benign/low-grade malignancy, confirmed postoperatively as a parenchymal adenomatoid tumor via immunohistochemistry. A 5-year prospective study assessed testicular function through serial measurements of serum hormones [follicle-stimulating hormone (FSH), luteinizing hormone (LH), testosterone, inhibin B (INHB), and anti-Müllerian hormone (AMH)] and bilateral testicular volume (TV). Our findings were as follows: Left TV exceeded age-adjusted norms by 2.1-fold (4.2 mL vs. 2.0 mL), contrasting with right TV at 0.5× mean (1.0 mL). AMH declined to a nadir (2.1 ng/mL) at 48 months, recovering to 3.8 ng/mL by 60 months. INHB reached minimal levels (45 pg/mL) at 6 months postresection, peaking at 128 pg/mL by study endpoint. FSH (1.2–1.5 IU/L) and testosterone (T, 0.15–0.18 ng/mL) remained prepubertal, while LH (0.3–0.5 IU/L) persisted near lower normative limits. This study demonstrates the progressive decline of Sertoli cell function in pediatric adenomatoid tumor survivors, detectable through longitudinal monitoring of INHB, AMH, and TV. The 2.1-fold compensatory hypertrophy in the affected testis suggests adaptive mechanisms requiring further investigation. Conservative resection guided by frozen section analysis prevents orchidectomy, while biomarker surveillance enables early detection of testicular dysfunction for timely intervention.

## Introduction

Testicular tumors have a relatively low incidence, accounting for 1% to 2% of all malignant tumors in males, with the majority being malignant (1). In 98% of the cases, malignant tumors of the testis are germ cell tumors, divided into pure seminomatous germ cell tumor (SGCT) and non-seminomatous GCT (NSGCT) in 60% and 40% of the cases, respectively (2). Therefore, the primary approach to treating testicular tumors is radical orchiectomy. The most common histological characteristics of these tumors before puberty are pure yolk sac tumors and teratomas (3, 4). Adenomatoid tumors are relatively uncommon benign tumors of mesothelial origin, usually occurring in the genital tract of both males and females. Previous literature reports have often presented individual cases, and there have been instances where organs were removed due to misdiagnosis as malignant tumors (5, 6). Currently, based on preoperative human chorionic gonadotropin (HCG), alpha-fetoprotein (AFP), and intraoperative rapid frozen section results, the option of partial preservation surgery for the affected part of the testis is chosen. Testicular adenomatoid tumor is an uncommon benign neoplasm of the testis, predominantly diagnosed in middle-aged individuals, while its occurrence in the pediatric population is exceedingly rare. In the US, approximately 10,000 new cases of testicular cancer are diagnosed annually. The mean age at diagnosis is 33 years, although 6% of cases occur in children and adolescents younger than 18 years and 8% occur in men older than 55 years (7). A comprehensive search of the PubMed database identified six case reports of adenomatoid tumors of the testis in adolescents (4 cases) and children (2 cases), with patients ranging in age from 14 months to 18 years at diagnosis. Therefore, case reports of adenomatoid tumors of the testis in children are even rarer, and there is a lack of relevant reports on monitoring and follow-up of testicular spermatogenic function. We report a case of partial excision surgery for intratesticular adenomatoid tumor in a 5.2-year-old child. What’s more, we are monitoring and doing a follow-up study of the testicular spermatogenic function. Written informed consent was obtained from the patient and his family for the publication of the present case.

## Case report

**Figure 1:**
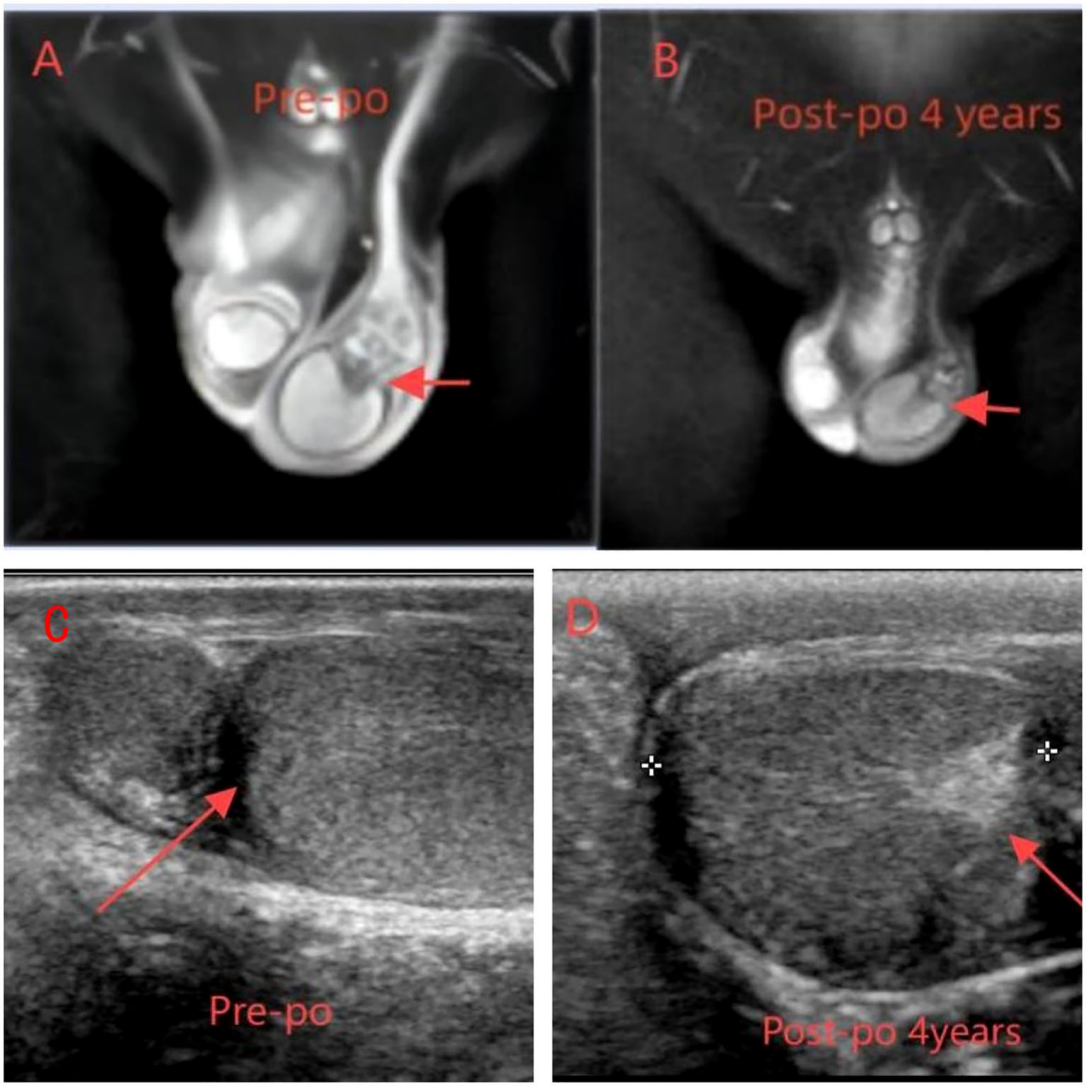
**(A, C)** MRI and ultrasound preoperation **(A)** On the outer side of the left testicle, a mixed signal mass of 13 × 14 mm was visible, with T2W1 sequence signals lower than the adjacent testicle. Mild enhancement was observed after contrast administration. **(C)** Enlargement of the left testis with slightly increased parenchymal echogenicity and globular thickening of the epididymal head. **(B, D)** MRI ultrasound postoperation **(B)** The left epididymal mass was less obvious than it was before the surgery. There was still a wedge-shaped low signal on the outer side of the testicle. **(D)** The left testis is enlarged and exhibits enhanced parenchymal echogenicity.

**Figure 2:**
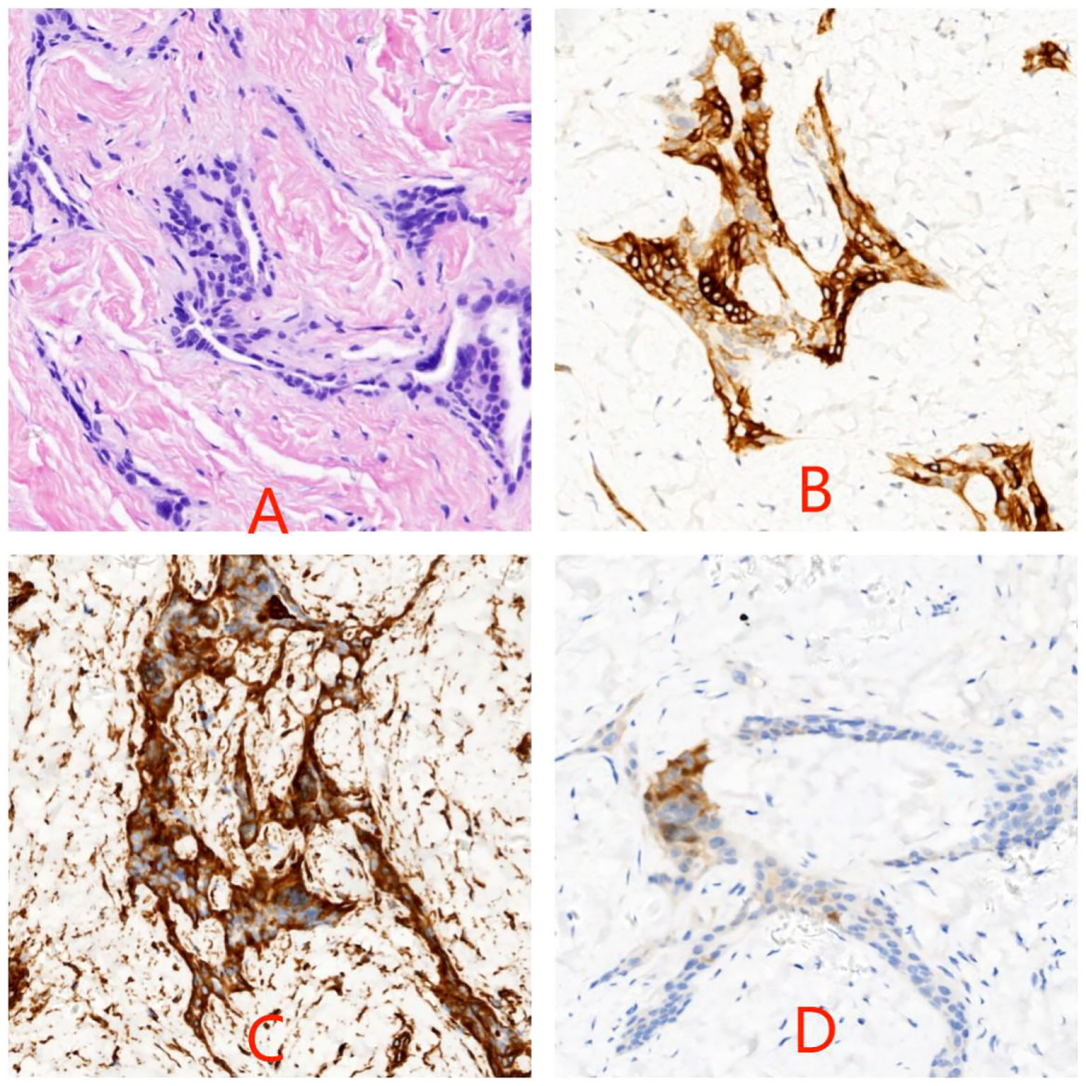
Photomicrograph of hematoxylin–eosin stain of testicular biopsy specimen in panel **(A)** Cytokeratin **(B)**, vimentin **(C)**, and calretinin **(D)** labelings are localized within the cytoplasm of tumor cells (original magnification ×200).

**Figure 3:**
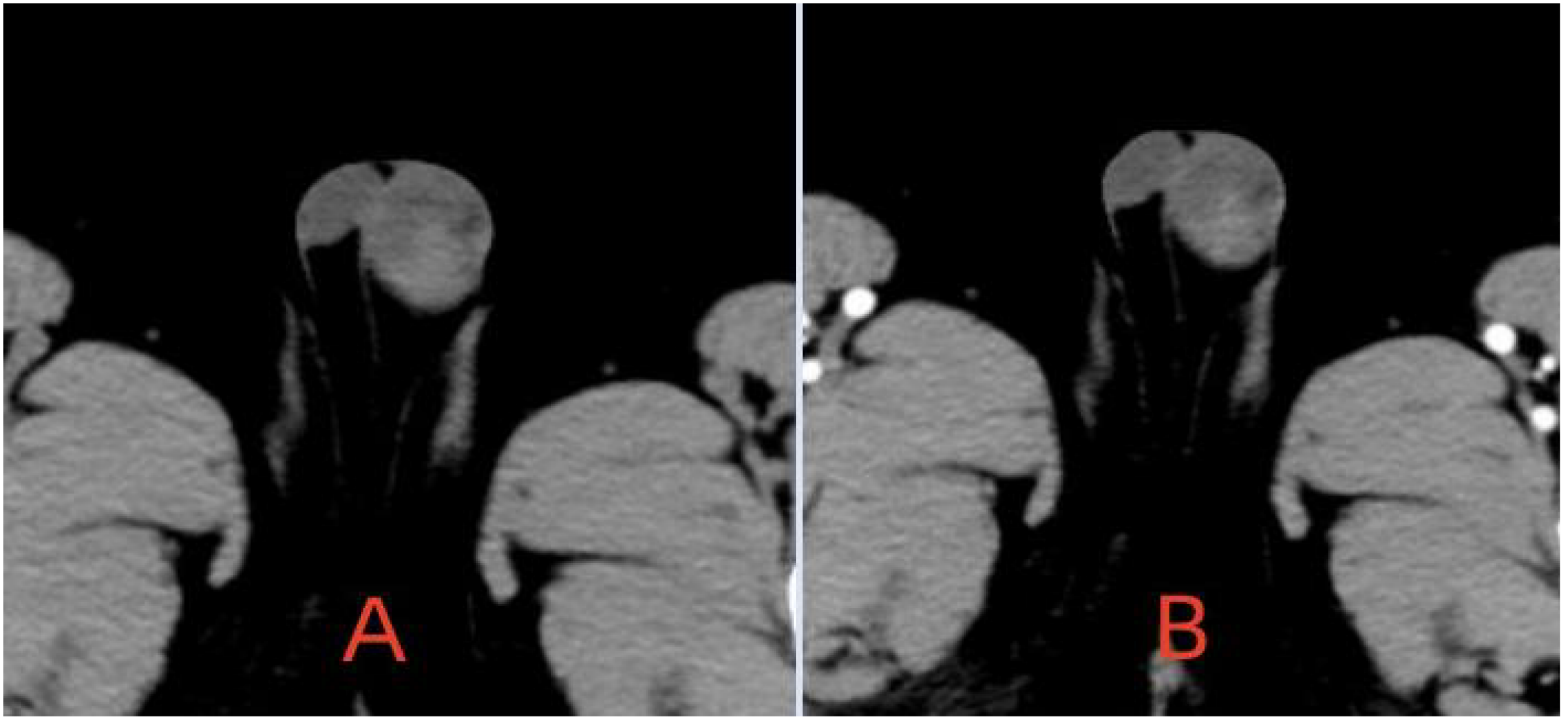
**(A)** CT scan. **(B)** CT enhancement showing the enhancement of some testicular tissue.

The patient is a 5.2-year-old boy who firstly had no other medical history and was admitted after the discovery of left scrotal enlargement for 1 month with no pain or other symptoms in June 2020. Scrotal ultrasound (Philips iU22 equipped with a high-frequency linear array probe) revealed the left testicle measuring 26×12×17 mm with slightly increased echogenicity, while the right testicle measured 12×7×11 mm (**Figure 1C**). MRI [Philips Achieva 1.5T (Netherlands) with a 16-channel abdominal coil] showed a significant enlargement of the left testicle, measuring 15×20×15 mm, with uneven internal signals. On the outer side of the left testicle, a mixed signal mass of 13×14 mm was visible, with T2W1 sequence signals lower than the adjacent testicle. Mild enhancement was observed after contrast administration (**Figure 1A**). Physical examination indicated an enlarged left testicle and a smaller right testicle. Both testicles were non-tender, and the cremasteric reflex was positive. The patient had no medical or surgical history. AFP and HCG tests were negative. Left testicular exploration was performed after admission, revealing unclear boundaries between the mass and testicular tissue. Partial tissue was excised for rapid frozen section pathological examination, which indicated a large proliferation of smooth muscle tissue with a few glandular structures. The cells were mild, suggesting a benign tumor or a low-grade malignant tumor. After informing the patient’s family of the diagnosis, a decision was made to perform partial excision surgery while preserving the testicle. Symptomatic and supportive therapy was given after surgery. One week later, the patient had a dry wound that healed well and was discharged. Postoperative pathology (**Figure 2**) and immunohistochemistry results (**Table 1**) confirmed the diagnosis of an adenomatoid tumor. We followed the patient and measured testicular volume by scrotal ultrasonography, monitoring endocrine function by measuring INHB, AMH, FSH, LH, and T after 3, 6, 12, 24, 36, 48, and 60 months. AFP and HCG tests were negative during follow-up at 5 years (**Table 2**), and ranges of INHB, AMH, LH, FSH, T serum levels according to ages for children are shown in **Table 3**. In the fourth year, we re-examined scrotal MRI and ultrasound (**Figures 1B, D**), which showed that the left epididymal mass was less obvious than it was before the surgery. There was still a wedge-shaped low signal on the outer side of the testicle, which had decreased in size compared with that before the surgery, raising suspicion of scar tissue or residual tumor. In the fifth year after surgery, we reviewed the enhanced CT, which showed that the left testis was significantly larger than the right side, about 27 × 20 mm, the internal density was uneven, the boundary was clear, and a part of testis enhancement was visible after the enhancement (**Figure 3**).

**Table 1:** Immunohistochemical staining of adenomatoid tumors.

Antibody									Staining intensity
CK (pan)	Vimentin	Ki-67	D2-40	Pax-8	CD99	CK7	Wilm’s tumor	Calretinin	+
Inhibin A	GATA-3	CK5	CK6	SMA	Desmin	SALL4	Melan-A (A103)	OCT-3/4	–

**Table 2:** Changes of serum AMH, INHB, TV, FSH, HLH, and T.

Time (month)	Testicular volume (mL)		INHB (pg/mL)	AMH(ng/mL)	FSH (mIU/mL)	LH (mIU/mL)	T (ng/dL)
	LTV	RTV					
0	2.776	0.484	23.3	54.35	0.95	0.10	7
3	2.814	0.486	25.8	48.6	0.82	0.12	6.9
6	2.862	0.491	19.6	42.5	1.28	0.21	7.2
12	3.132	0.586	23.16	48.6	1.31	0.16	8.1
24	3.062	0.642	28.4	47.3	1.26	0.12	6.2
36	3.184	0.785	22.6	45.72	1.36	0.18	9.4
48	3.562	0.825	26.2	39.79	1.45	0.3	7.6
60	3.926	0.926	31.4	43.87	1.92	0.13	8.9

INHB (pg/mL), inhibin B; AMH (ng/mL^3^), anti-Müllerian hormone; LTV, left testicular volume; RTV, right testicular volume; FSH, serum follicle-stimulating hormone; LH, serum luteinizing hormone. Testicular volume (mL) = π/6 × (length × width × height).

**Table 3:** Ranges of INHB, AMH, LH, FSH, and T serum levels according to ages for children.

Ages	INHB (pg/mL)	Ages	AMH (ng/mL)	Ages	LH (mIU/mL)	FSH (mIU/mL)	Ages (years)	T (ng/dL)
1–7 days	49.2–133.7	0–14 days	35–140	0–3 months	0.78–4.26	0.76–4.1	0–2	<14–1242
7 days–2 months	60.8–240.1	15 days–6 months	55–210	3–12 months	0–2.86	0.54–2.16	2–4	<14–57
3–4 months	270.2–348.7	6 months–2 years	85–320	1–2 years	0–0.32	0.31–1.49	4–6	<14–48
5–11 months	149.3–240.9	2–9 years	55–250	2–9 years	0–0.45	0.18–1.92	6–8	<14–40
1–2 years	67.8–128.4	9–18 years	4–200	>9 years	0–1.77	1.3–3.22	8–10	<14–60
3–4 years	52.4–93	adults	3–18				10–12	<14–207
5–12 years	21.6–64.4						12–14	<14–564

## Discussion

Adenomatoid tumors are the most common paratesticular neoplasms and account for approximately 30% of all paratesticular masses (8). Most adenomatoid tumors are asymptomatic and present as small, solid, non-tender masses and having a lack of specific clinical and radiological features (8). Thus, importance lies in the preoperative differential diagnosis with malignant tumors of the testis. Ultrasound is the initial imaging, but MRI correlation has the utility of determining the origin of the mass. The purpose of treating these masses is to prevent unnecessary orchiectomy, thereby preserving fertility and testosterone production. In this regard, preoperative assessing of serum tumor markers like alpha-fetoprotein and beta-HCG and intraoperative frozen section analysis might help in excluding a malignant lesion. It helps the urological surgeon’s intraoperative decision to either excise the local lesion (and risk leaving a malignancy) or to perform radical orchiectomy. Currently, there is no relevant report on whether adenomatoid tumors of the testis affect testicular endocrine function (spermatogenic cells, supporting cells, and interstitial cells) during prepuberty. Waqas Amin (9) suggests its origin to be mesothelial even though these tumors occur in hormone-responsive organs but no hormone receptors have been discovered in adenomatoid tumors, and there is no proof that these are hormone sensitive. However, Sharpe (10) considered that it is possible that the secreted products of testicular stromal cells may affect the synthesis of Sertoli intracellular inhibin. We believe that patients who undergo testicular tumor surgery in the prepubertal period should be followed up and monitored for INHB, AMH, FSH, LH, testosterone, and TV after surgery to assess testicular spermatogenic function, thereby evaluating testicular function during puberty.

Gonadotropin-releasing hormone (GnRH) secreted by the human hypothalamus stimulates the synthesis and secretion of follicle-stimulating hormone (FSH) and luteinizing hormone (LH) from the anterior pituitary gland. FSH acts on the seminiferous tubules to initiate meiosis in spermatogonia and promote early spermatogenic cell development. It also enhances Sertoli cell secretion of androgen-binding protein (ABP), thereby facilitating sperm production and maturation. In parallel, the pulsatile release of LH stimulates testosterone synthesis and secretion by Leydig cells. This testosterone diffuses into the seminiferous tubules to support spermatogenesis. The complete spermatogenic process requires coordinated actions of both FSH-mediated Sertoli cell activation and LH-induced testosterone production from Leydig cells. Inhibin B (INHB), predominantly secreted by Sertoli cells, exerts negative feedback inhibition on FSH secretion. Elevated serum FSH levels typically indicate primary testicular dysfunction, where impaired Sertoli cell function reduces INHB production, consequently diminishing its inhibitory effect on pituitary gonadotropins. The degree of FSH elevation correlates with the severity of damage to the Sertoli cell-seminiferous tubule complex. Progressive testicular impairment involving Leydig cell dysfunction manifests as subsequent LH level elevation (11). These findings collectively demonstrate that serum concentrations of FSH, LH, and INHB serve as critical biomarkers for evaluating testicular spermatogenic function.

During the prepubertal period, spermatogenic cells and interstitial cells in the testis are relatively dormant. Sertoli cells are a major component of testicular volume during the embryonic period and prepubertal period. AMH and INHB in the prepubertal period are entirely produced by testicular supporting cells (12, 13). The secretion levels of anti-Müllerian hormone (AMH) and inhibin B (INHB) represent the function of supporting cells, indirectly reflecting testicular function in prepubertal children (14). Karaoglan’s (12) study suggests that serum AMH testing can serve as a reliable and practical biomarker for evaluating testicular function in male adolescents with hypogonadism. INHB is more sensitive than testicular volume, testosterone, and other factors in the clinical assessment of prepubertal testicular function and may be the most valuable endocrine marker to understand future spermatogenic conditions (15, 16). However, its diagnostic value is not superior to follicle-stimulating hormone (FSH) (17). Around the age of 10 years, as the volume of testicular and serum testosterone increases, serum statibin B concentrations begin to rise, reaching the Tanner phase II levels (18). Blevrakis (19) found that children with varicocele in Tanner III–IV had decreased inhibin B levels. Therefore, it seems that serum inhibin B levels can suggest that the age group from 10 to 13 years may be the window period for intervention. A similar view was also suggested by Goulis et al. (20). Yasenjiang Abudoula et al. reported that serum AMH and serum INHB levels can be used as a reliable indicator for assessing testicular function in patients with cryptorchidism and can evaluate testicular function recovery after testicular fixation (21).

When testicular lesions result in damage to spermatogenic cells, dynamic alterations in serum levels of follicle-stimulating hormone (FSH), luteinizing hormone (LH), and inhibin B (INHB) can be observed. Elevated serum FSH levels are primarily attributed to primary testicular dysfunction, which impairs the secretory capacity of Sertoli cells (rather than Leydig cells) to produce INHB. This hormonal deficiency subsequently diminishes the negative feedback inhibition on pituitary gonadotropin release. The magnitude of FSH elevation correlates with the severity of damage to the Leydig cell-seminiferous tubule axis. Progressive testicular impairment further disrupts interstitial cell function, manifested by subsequent LH level elevation. Consequently, the combined analysis of serum FSH, LH, and INHB concentrations serves as a clinically valuable indicator for evaluating spermatogenic competence and testicular endocrine function. Seminiferous tubules constitute 98% of testicular volume, making testicular size a strong indicator of spermatogenic status (22). These tubules are highly sensitive to ischemic or infectious insults, which may cause spermatogenic cell depletion, Sertoli cell dysfunction, and subsequent tubular atrophy. Such pathological changes lead to reduced testicular volume and impaired or lost spermatogenic capacity (23).

Therefore, we conducted early testicular function monitoring in patients undergoing testicular tumor surgery, and timely abnormal detection and timely intervention. Through a 5-year follow-up of this case, we found that both testes developed, but the left testicular volume was about 2.1 times the mean volume at this age, but the right testis just was 0.5 times (23). AMH reached its lowest value in the 48th month and rose in the 60th month; INHB was at the lowest value in the 6th postoperative month, reaching the highest value in the 60th month. LH levels were lower-normal, and FSH and T were within the prepubertal normal range. The longitudinal endocrine and volumetric assessment protocol implemented in this study represents a landmark approach in pediatric testicular tumor surveillance. Our 5-year hormonal profiling uniquely captures the dynamic trajectory of Sertoli cell function recovery, particularly evidenced by the INHB resurgence from 45 pg/mL to 128 pg/mL and AMH rebound from 2.1 ng/mL to 3.8 ng/mL. These quantitative biomarkers, when correlated with the persistent 2.1-fold testicular hypertrophy (4.2 mL vs. age-adjusted 2.0 mL), provide unprecedented insights into Sertoli cell hyperplasia and mediate the restoration of hormonal profiles, subsequently driving testicular compensatory hypertrophy following conservative resection. Another hypothesis is that the observed testicular enlargement in this case may be attributed to residual adenomatoid tissue with persistent expansile growth despite the tumor’s benign histology. Notably, the stability of reproductive hormones does not preclude subclinical architectural damage, as evidenced by volumetric asymmetry. The implementation of longitudinal hormonal and volumetric surveillance provides critical insights into testicular functional integrity, with serum biomarkers (INHB, AMH, FSH, and LH) and high-resolution ultrasonographic volumetry collectively serving as indispensable modalities for monitoring gonadal homeostasis. This dual-parameter assessment paradigm enables early detection of Sertoli cell dysfunction (evidenced by declining INHB/AMH trajectories) and compensatory adaptive responses (manifested by testicular hypertrophy exceeding 200% of age-adjusted norms), thereby establishing a functional–anatomic correlation essential for optimizing pediatric testicular tumor management. According to this result, we suspected that intrapinoestiticular adenomatoid tumor had an effect on Sertoli cells development, and then decreased testicular endocrine function. For the next 5 years, we will continue to monitor TV, INHB, AMH, and sex hormones to assess their testicular function. Our longitudinal study challenges the traditional “wait-and-see” approach for pediatric testicular tumors by demonstrating that even histologically benign lesions (e.g., adenomatoid tumors) can insidiously impair Sertoli cell–endocrine crosstalk. The delayed recovery of AMH (peaking at 60 months postoperation) underscores the necessity of extending follow-up beyond puberty to detect latent dysfunction. Incorporating TV–INHB–AMH triparametric surveillance into clinical protocols may revolutionize risk stratification and timely intervention for preserving fertility in this vulnerable population. Further research needs to expand the sample, conduct Tanner staging for each case, and group continuous TV, INHB, AMH, and sex hormone levels for surgery or conservative treatment, so as to observe whether TV, INHB, AMH, and sex hormone levels can well evaluate the function of testicular supporting cells in adolescents and become one of the indications for surgery.

## Conclusion

Our case advocates for testis-sparing surgery with frozen section guidance in pediatric testicular tumors, minimizing unnecessary orchidectomy. Longitudinal monitoring of INHB, AMH, and testicular volume revealed subclinical dysfunction despite normal sex hormones, necessitating yearly hormonal and ultrasound surveillance until late adolescence. Integrating organ preservation with protocolized monitoring optimizes oncologic and functional outcomes in children.

## Data Availability

The original contributions presented in the study are included in the article/supplementary material. Further inquiries can be directed to the corresponding authors.
